# No preclinical rationale for IGF1R directed therapy in chondrosarcoma of bone

**DOI:** 10.1186/s12885-016-2522-8

**Published:** 2016-07-14

**Authors:** Elisabeth F. P. Peterse, Arjen H. G. Cleven, Yvonne De Jong, Inge Briaire-de Bruijn, Jonathan A. Fletcher, Erik H. J. Danen, Anne-Marie Cleton-Jansen, Judith V. M. G. Bovée

**Affiliations:** Department of Pathology, Leiden University Medical Center, Leiden, The Netherlands; Department of Pathology, Brigham and Women’s Hospital and Harvard Medical School, Boston, Massachusetts USA; Division of Toxicology, Leiden Academic Center for Drug Research, Leiden University, Leiden, The Netherlands

**Keywords:** Chondrosarcoma, IGF1R signalling, Insulin-like growth factor, OSI-906, Sarcoma

## Abstract

**Background:**

Chondrosarcoma is a malignant cartilage forming bone tumour for which no effective systemic treatment is available. Previous studies illustrate the need for a better understanding of the role of the IGF pathway in chondrosarcoma to determine if it can be a target for therapy, which was therefore explored in this study.

**Methods:**

Expression of mediators of IGF1R signalling and phosphorylation status of IRS1 was determined in chondrosarcoma cell lines by qRT-PCR and western blot. The effect of activation and inhibition of IGF1R signalling on downstream targets was assessed by western blot. Ten chondrosarcoma cell lines were treated with OSI-906 (IGF1R and IR dual inhibitor) after which cell proliferation and migration were determined by a viability assay and the xCELLigence system, respectively. In addition, four chondrosarcoma cell lines were treated with a combination of doxorubicin and OSI-906. By immunohistochemistry, IGF1R expression levels were determined in tissue microarrays of 187 cartilage tumours and ten paraffin embedded cell lines.

**Results:**

Mediators of IGF1R signalling are heterogeneously expressed and phosphorylated IRS1 was detected in 67 % of the tested chondrosarcoma cell lines, suggesting that IGF1R signalling is active in a subset of chondrosarcoma cell lines. In the cell lines with phosphorylated IRS1, inhibition of IGF1R signalling decreased phosphorylated Akt levels and increased IGF1R expression, but it did not influence MAPK or S6 activity. In line with these findings, treatment with IGF1R/IR inhibitors did not impact proliferation or migration in any of the chondrosarcoma cell lines, even upon stimulation with IGF1. Although synergistic effects of IGF1R/IR inhibition with doxorubicin are described for other cancers, our results demonstrate that this was not the case for chondrosarcoma. In addition, we found minimal IGF1R expression in primary tumours in contrast to the high expression detected in chondrosarcoma cell lines, even if both were derived from the same tumour, suggesting that *in vitro* culturing upregulates IGF1R expression.

**Conclusions:**

The results from this study indicate that the IGF pathway is not essential for chondrosarcoma growth, migration or chemoresistance. Furthermore, IGF1R is only minimally expressed in chondrosarcoma primary tumours. Therefore, the IGF pathway is not expected to be an effective therapeutic target for chondrosarcoma of bone.

**Electronic supplementary material:**

The online version of this article (doi:10.1186/s12885-016-2522-8) contains supplementary material, which is available to authorized users.

## Background

Chondrosarcoma is the second most common primary bone malignancy in humans [1] and represents a heterogeneous collection of cartilage forming tumours with different outcomes depending on subtype and histological grade. Conventional central chondrosarcoma, arising centrally in the medulla of bone, accounts for ~85 % of the cases and can be histologically divided into 3 grades [1]. Sixty-one percent of these tumours are classified as atypical cartilage tumour (ACT) (previously known as grade I), for which first line treatment is curettage with local adjuvant treatment, resulting in a 5 year survival rate of 83 %. Grade II (36 %) and grade III (3 %) tumours are more prone to metastasize and have a combined 5 year survival rate of 53 % [1–3]. These tumours are treated with en bloc resection. Dedifferentiated chondrosarcoma is a highly malignant variant with an overall survival rate of 7 ~ 24 % [4]. Mesenchymal chondrosarcoma is a rare aggressive subtype, in which distant metastasis can be identified even after 20 years [5]. It has a 10 year survival rate between 44 and 54 % [6, 7]. Chondrosarcoma patients with unresectable disease, due to tumour location, tumour size or extensive metastatic disease, have a 5 year survival of only 2 % [8]. Although chondrosarcoma is known for its resistance to chemo- and radiotherapy, it was recently described that patients with inoperable disease treated with doxorubicin-based chemotherapy have a 3 year survival rate of 26 % versus 8 % in patients who did not receive systemic treatment [8] and chemotherapy sensitivity differed between the chondrosarcoma subtypes [9]. However, it is clear that overall efficacy of chemotherapy is limited. So far, the discovered genetic alterations and pathways involved in chondrosarcoma (reviewed in [10] and [11]) have not resulted in new treatment regimes*.* Therefore, further unravelling of critical signalling pathways in chondrosarcoma is essential to identify new therapeutic targets.

One pathway which has been implicated in chondrosarcoma proliferation is the IGF pathway. The IGF pathway has two closely related ligands: IGF1 and IGF2 [12]. When a ligand binds to the IGF1 receptor (IGF1R), this receptor forms homodimers or hybrid receptors with the insulin receptor (IR). The resulting autophosphorylation of the receptor recruits the insulin receptor substrate (IRS) to the membrane causing subsequent downstream activation of the PI3K/Akt/mTOR pathway and the Ras/Raf/MEK signalling pathway, which are known to be driver pathways in cancer [12]. IGF2R functions to decrease the availability of IGF2 to IGF1R [12].

IGF1R can be the upstream receptor that is responsible for the well known activation of the PI3K/Akt/mTOR pathway, the Src-pathway and the Ras/Raf/MEK pathway in (a subset of) chondrosarcoma cell lines and primary cultures [13–17]. In a heterogeneous group of sarcoma patients, a combination of an IGF1R antibody and mTOR inhibitor has been shown to have clinical activity but the level of IGF1R expression was not predictive for response [18]. Takigawa et al. demonstrated that cells of a clonal human chondrosarcoma-derived chondrocyte cell line produce IGF ligands and express IGF1R and IGF2R [19]. Seong et al. and Matsumari et al. described that IGF1 increases cell proliferation in a Swarm-rat chondrosarcoma model [20, 21]. Interestingly, Ho et al. described that IGF binding protein 3 (IGFBP3), which binds the IGF ligands thereby inhibiting their interaction with the IGF receptors, decreases with increasing histological grade of chondrosarcoma [22]. In addition, Wu et al. demonstrated that IGF1 induced migration of chondrosarcoma cell lines which could be blocked by an IGF1R antibody [23]. Recently, functional profiling of receptor tyrosine kinases in chondrosarcomas revealed active IGF1R signalling in one out of five chondrosarcoma cell lines [13].

These above mentioned studies illustrate the need for a better understanding of the role of IGF1R signalling in chondrosarcoma to determine if it is a convincing target for therapy. Because chondrosarcoma is a very heterogenous disease, it is possible that the IGF1R directed therapy is only effective in a subset of patients. Hence, we used our large chondrosarcoma cell line panel, including three grade 2 and three grade 3 conventional chondrosarcomas, three dedifferentiated chondrosarcomas and one mesenchymal chondrosarcoma cell line. We analyzed expression levels of IGF1R and other important mediators of IGF1R signalling and determined the effect of IGF1R inhibitors. Our results indicate that the IGF pathway is not important for chondrosarcoma growth as IGF1R inhibition did not demonstrably impact chondrosarcoma cell line proliferation, migration and chemoresistance. In addition, IGF1R expression is low/absent in chondrosarcoma primary tumours in contrast to chondrosarcoma cell lines. This illustrates that there is limited preclinical rationale for using IGF1R inhibitors for the treatment of chondrosarcoma of bone.

## Methods

### Compounds

The IGF1R inhibitors OSI-906, NVP-ADW742 and GSK1838705A were purchased from Selleck Chemicals LCC (see Additional file 1: Table S1 for properties) and dissolved in DMSO in a concentration of 10 mM. The IGF1R inhibitors were tested in concentrations up to 1 μM as it was demonstrated previously that higher concentrations lead to an aspecific toxic response [24]. Recombinant human IGF1 (PeproTech) was used in a concentration of 50 ng/ml [25]. Doxorubicin was obtained from the in-house hospital pharmacy in a 0.9 % NaCl solution, and used in a concentration range of 1–100 nM.

### Cell culture

The conventional chondrosarcoma cell lines JJ012 [26], SW1353 (ATCC), CH2879 [27], OUMS27 [28], L835 [29] and CH3573 [30], as well as the dedifferentiated chondrosarcoma cell lines L3252B [29], NDCS1 [31], and L2975 [29] were cultured in RPMI 1640 (Gibco, Invitrogen) supplemented with 1 % Glutamax (Gibco 35050, Invitrogen), 1 % penicillin/streptomycin (PS) (100U/mL) (Gibco, Invitrogen) and 10 % (JJ012, SW1353, CH2879, NDCS1, L2975) or 20 % (L835, L3252B, OUMS27, CH3573) heat-inactivated Fetal Bovine Serum (FBS) (F7524, Sigma-Aldrich). MCS170 (Mesenchymal chondrosarcoma) and TC-32 (Ewing Sarcoma, [32]) were cultured in IMDM (Gibco, Invitrogen) with 1 % PS with respectively 15 and 10 % FBS. The cells were grown at 37 °C with 5 % CO_2_ in a humidified incubator. Mycoplasma tests were performed regularly. Identity of cell lines was confirmed using STR profiling with the CELL ID^™^ system (Promega Benelux BV).

### qRT-PCR

RNA isolation and cDNA synthesis was performed as described [33]. To determine the expression levels of IGF1, IGF1R, IGF2, IGF2R, IGFBP3 and IR, a standard quantitative reverse transcriptase PCR (qRT-PCR) was performed as described previously [34]. Primers (Additional file 2: Table S2) were designed using primer3 software (http://bioinfo.ut.ee/primer3/). To correct for the amount of cDNA input, gene expression levels were normalized using the expression levels of CYPa and CPSF6 [35, 36]. ΔΔCq values below 0.01 were considered negative. All qRT-PCRs were optimized on control tissue as indicated in Additional file 2: Table S2.

### Immunoprecipitation and Western Blotting

Western blotting was performed as described previously [14]. Per sample, 20 μg of protein was loaded on SDS-PAGE gels. Rabbit antibodies against IGF1R (#3018), IR (#3025),IRS1 (#2382) and Phospho-S6 Ribosomal Protein (Ser235/236) (2 F9) (#4856) all diluted 1:1000, were obtained from Cell Signaling. Phospho-Akt (Ser473) (#9271), diluted 1:2000 was also obtained from Cell Signaling. Rabbit polyclonal antibody against phospho-IRS1 (Y612, 1:1000) was purchased from Biosource, Invitrogen. Monoclonal Anti-MAP Kinase, Activated (Diphosphorylated ERK-1&2) was obtained from Sigma (M8159) and diluted 1:2000. A mouse monoclonal antibody against α-tubulin (1:3000) (Abcam) was used as a loading control. Secondary antibodies were horseradish peroxidase (HRP) conjugated polyclonal goat-anti-rabbit IgG for components for IGF1R, IR, IRS1, phospho-IRS1, pAkt and pS6, and HRP conjugated polyclonal goat-anti-mouse for α-tubulin and diphos. ERK-1&2 (both 1:3000, BD Transduction Laboratories). Immunoprecipitation (IP) for IRS1 was performed according to the manufacturer’s instruction. In short, cells were harvested at ±80 % confluence using the Cell lysis buffer (Cell Signaling) to which the PhosSTOP (REF 04906837001) and the Protease Inhibitor Cocktail Tablets (REF 11697498001) were added (Roche). The IRS1 antibody (1:50) was added to 200 μl lysate at 1 mg/ml and rotated over night at 4 °C, followed by 30 min incubation with 40 μl protein A magnetic beads (Cell Signaling). After washing using a magnetic separation rack, the pellet was suspended in 3x SDS sample buffer (containing 2-Mercaptoethanol). The sample was loaded on an SDS-PAGE gel and western blotting was performed as described above. TC-32 was used to optimize the protocol and was included as a positive control.

### Proliferation assay

In all cell viability experiments, the cell lines were plated in triplicate at a density of 3000 to 10000 cells per well depending on the growth rate. For the positive control (Ewing sarcoma cell line TC-32) the 96 well plates were coated with gelatine. After the cells were allowed to adhere overnight, the IGF1R inhibitors were added in their corresponding concentrations. In addition, we determined the effect of OSI-906 when IGF1 (50 ng/ml) was added to the medium. For the combination treatment of doxorubicin and OSI-906, both inhibitors were added at the same time. Because JJ012 and SW1353 are relatively more sensitive to doxorubicin, JJ012 and SW1353 were treated with 0, 1 nM, 10 nM or 100 nM while CH2879 and OUMS27 were treated with 0, 10 nM, 50 nM and 100 nM doxorubicin. These concentrations of doxorubicin were combined with DMSO, 0.1 μM, 0.5 μM or 1 μM OSI-906. After 72 h of incubation, cell viability was measured using the WST-1 reagent (Roche) (single treatment with OSI-906) or PrestoBlue Cell Viability Reagent (Promega Benelux BV) (single treatment with NVP-ADW742 and GSK1838705A and combinations with IGF1 and doxorubicin) according to the manufacturer’s instructions. Colorimetric values in the plates were subsequently measured using a Wallac 1420 VICTOR2 (Perkin Elmer). Data were analysed in Graphpad Prism 5.0 (www.graphpad.com). The results shown are representative results from at least three independent experiments.

### Migration assay

The real-time cell analyser xCELLigence system (Roche) based on cell-electrode subtract impedance detection technology [37] was used to study the effect of IGF1R/IR inhibition on migration as previously described [16]. In short, cell lines were added at a density of 80.000 per well in the upper chamber of the Cell Invasion and Migration (CIM) plates in serum-free RPMI medium containing 0, 100 nM or 1 μM OSI-906. The lower chambers were filled with RPMI medium supplemented with 20 % FBS. The software calculated the Cell index, which was set at 1.0 migration at the last measurement.

### Immunohistochemistry on tissue microarrays

The specificities of two IGF1R antibodies (#3018 and #3027, Cell Signaling) were compared by western blot (as described above) and immunohistochemistry on colon tissue (as described in [38]). The most specific antibody was selected to determine the IGF1R expression in 5 enchondromas, 7 osteochondromas, 71 central conventional chondrosarcomas, 34 peripheral conventional chondrosarcomas, 32 dedifferentiated chondrosarcomas, 18 mesenchymal chondrosarcomas and 20 clear cell chondrosarcomas by using previously constructed and described tissue microarrays (TMAs) [16, 39]. Slides were scored by an experienced pathologist (AHGC) as either positive or negative.

## Results

### IGF pathway members are expressed in a subset of chondrosarcoma cell lines

**Fig. 1:**
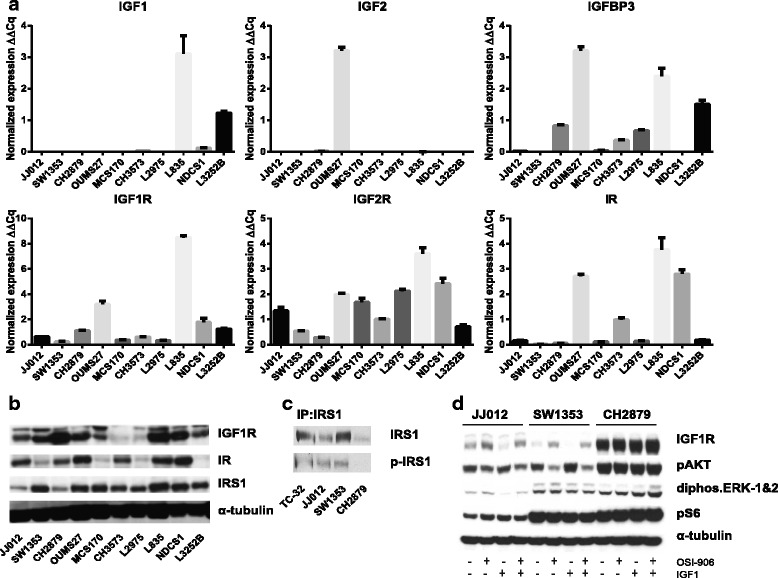
**a-b** qRT-PCR and western blot analyses, respectively, reveal heterogeneous expression of IGF pathway members in chondrosarcoma cell lines. **c** Immunoprecipitation with IRS1 followed by a western blot for phospho-IRS1 reveals pathway activity in two out of three chondrosarcoma cell lines tested. **d** Evaluation of IGF1R downstream targets reveals an effect of OSI-906 on IGF1R and pAkt but not on pS6 and disphosphorylated ERK-1&2. Cell lines were treated for 72 hours with DMSO, 1 μM OSI-906 and/or 50 ng/ml IGF1

Using qRT-PCR analyses, we demonstrate that all cell lines express the receptors IGF1R, IGF2R and IR (Fig. 1a). However, expression levels are highly variable as L835, OUMS27 and NDCS1 have a relatively high expression of the three receptors as compared to the other cell lines. For the IGF1R and the IR, we correlated the mRNA expression levels to levels of protein expression (Fig. 1b). mRNA expression of the ligand IGF1 is restricted to four out of ten chondrosarcoma cell lines, with the highest expression in L835. Strikingly, IGF2 expression in OUMS27 is very high, comparable to the expression levels in a human placenta (data not shown). IGFBP3 mRNA expression is detected in 8 out of 10 chondrosarcoma cell lines. In addition, western blot analyses revealed protein expression of IRS1 in all cell lines, although expression levels are again variable amongst the different cell lines (Fig. 1b).

### IGF1R signalling is active in a subset of chondrosarcoma cell lines

To determine IGF pathway activity, the phosphorylation status of IRS1 was determined in three chondrosarcoma cell lines. Immunoprecipitation for IRS1 followed by western blot analyses with a phospo-IRS1 antibody revealed the presence of phosphorylated IRS1 in JJ012 and SW1353, but not in CH2879 (Fig. 1c). This demonstrates that IGF1R signalling is active in a subset of chondrosarcoma cell lines. Furthermore, only in the cell lines in which phosphorylated IRS1 was detected, phosphorylated Akt levels were decreased and IGF1R levels were increased by OSI-906 (dual IGF1R and IR inhibitor) treatment (Fig. 1d). However, phosphorylated S6, located downstream of Akt, and diphosphorylated ERK-1&2 were unaffected, suggesting that activity of the downstream targets is not dependent on IGF1R signalling.

### Viability and migration of chondrosarcoma cell lines is not affected by IGF1R inhibition

**Fig. 2:**
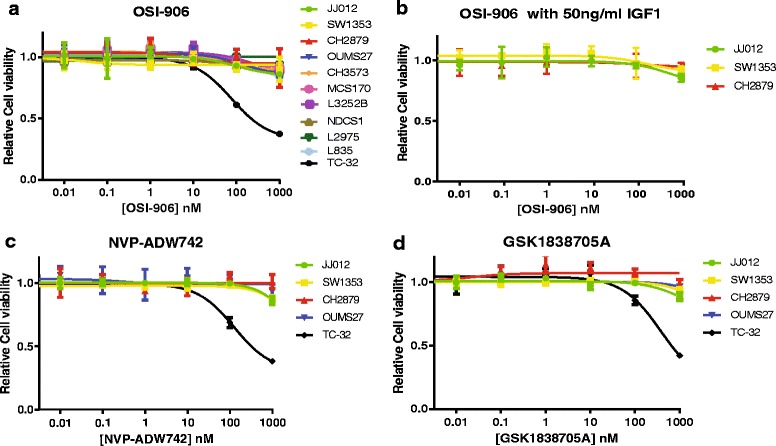
Relative cell viability of 72 hours of treatment with IGF1R/IR inhibitors. **a**-**b** OSI-906 does not inhibit chondrosarcoma cell viability, even in the presence of IGF1. **c**-**d** IGF1R inhibitors NVP-ADW742 and GSK1838705A do not inhibit chondrosarcoma cell viability

Treating our full chondrosarcoma cell line panel for 72 h with concentrations from 0.01 to 1000 nM of OSI-906 revealed that chondrosarcoma cell viability was not affected by inhibition of the IGF pathway, whereas the positive control cell line TC-32 (Ewing sarcoma) showed dose-dependent decrease of cell viability (Fig. 2a). Furthermore, addition of IGF1 to the medium did not increase cell proliferation (Additional file 3: Figure S1) nor sensitivity to OSI-906 (Fig. 2b) in three chondrosarcoma cell lines tested. In addition, four chondrosarcoma cell lines and the Ewing sarcoma cell line were treated with two other IGF1R/IR inhibitors (NVP-ADW742 and GSK1838705A) to determine if alternative targeting showed similar effects on cell viability (Fig. 2c and d). Indeed, the results were highly comparable, demonstrating that the IGF pathway is not essential for chondrosarcoma cell viability.

**Fig. 3:**
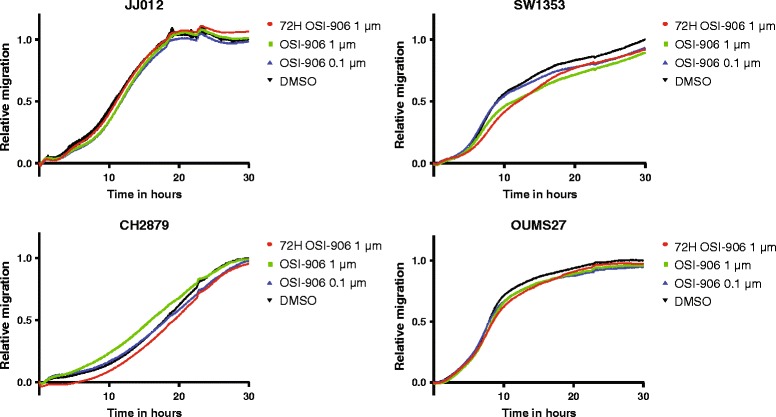
OSI-906 does not inhibit migration of four chondrosarcoma cell lines. 72H OSI 1 μM: cells were treated for 72 hours with OSI-906 before experimental onset

By adding OSI-906 to the upper chamber of the CIM plates (xCELLigence), we demonstrated that IGF1R signalling was not essential for the migration of JJ012, SW1353, CH2879 and OUMS27 (Fig. 3). To exclude the possibility that the absence of an effect of OSI-906 on chondrosarcoma cell migration was caused by an insufficient treatment duration, the cell lines were treated with 1 μM OSI-906 for 72 h before the onset of the experiment in one experimental condition. However, even after this pretreatment, chondrosarcoma cell line migration was not influenced by IGF1R/IR inhibition, illustrating that the IGF pathway does not play a role in chondrosarcoma cell migration.

### The IGF pathway is not involved in chondrosarcoma chemoresistance

**Fig. 4:**
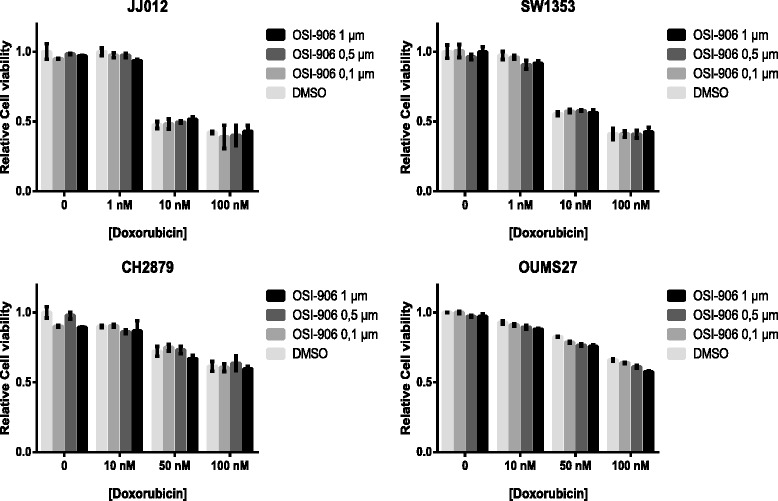
Relative cell viability after 72 hours of combination treatment with OSI-906 and Doxorubicin. OSI-906 does not sensitize the cells to doxorubicin

Because IGF1R signalling has been implicated in chemoresistance [40], four chondrosarcoma cell lines were treated with a combination of OSI-906 and doxorubicin. Although doxorubicin inhibited cell viability in a dose dependent manner, IGF1R/IR inhibition did not increase this cytotoxicity in any of the cell lines (Fig. 4). This indicates that the IGF pathway is not involved in chondrosarcoma chemoresistance.

### IGF1R is not strongly expressed in uncultured cartilage tumours

**Fig. 5:**
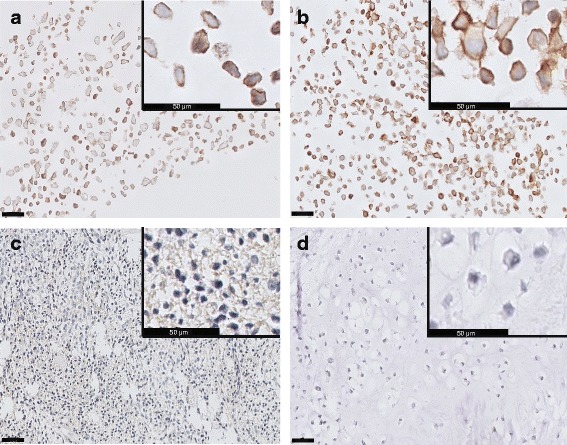
IGF1R expression is high in chondrosarcoma cell lines but low in primary tumours. **a** IGF1R expression in chondrosarcoma cell line JJ012. **b** and **d** IGF1R expression in L835 cell line and primary tumour, respectively. **c** a conventional chondrosarcoma sample that was classified as weak IGF1R staining. Black bars represent 50 μm

To elucidate the discrepancy between the observed protein expression of IGF1R in cell lines and the absence of an effect of IGF pathway inhibition, we assessed IGF1R expression in clinical tumour samples versus cell lines that were formalin-fixed and paraffin-embedded (FFPE). IGF1R antibody #3018 was selected for these immunohistochemical stainings as both the positive immunohistochemical control and the control western blot demonstrated its high specificity compared to the IGF1R antibody #3027 (Additional file 4: Figure S2). Immunohistochemistry confirmed the western blot evidence of IGF1R expression (Fig. 1b), with membranous expression of IGF1R shown in ten cell lines, and IGF1R expression levels were comparable in western blot and immunohistochemistry evaluations (Fig. 5a-b). In contrast, the primary tumours were either completely negative (66 %) or showed very weak staining (34 %) for IGF1R (Fig. 5c, Table 1). To exclude the possibility that the discrepancy between the primary tumours and the cell lines was due to tissue handing procedures, we included a colon tissue sample that was decalcified by 20 % formic acid for 2 days which stained positive thereby excluding an effect of the decalcification procedure (Additional file 5: Figure S3). To further study the difference in IGF1R expression between primary tumours and cell lines, we stained the primary tumours corresponding to the cell lines L835 [29], CH2879 [27], L3252B [29] and L2975 [29]. Strikingly, the primary tumours were either completely negative (L835, CH2879) or showed weak staining (L3252B, L2975) for IGF1R (Fig. 5d, Additional file 6: Figure S4). This suggests that chondrosarcoma cells upregulate IGF1R upon prolonged culturing.

**Table 1:** Immunohistochemistry demonstrates only weak IGF1R expression in uncultured cartilage tumours

Diagnose		Weak IGF1R positivity
Enchondroma		3/5 (60 %)
Osteochondroma		2/7 (29 %)
Central Conventional chondrosarcoma		32/71 (45 %)
	Grade I	8/28 (29 %)
	Grade II	15/29 (51 %)
	Grade III	9/14 (64 %)
Peripheral Conventional chondrosarcoma		14/34 (41 %)
	Grade I	8/21 (38 %)
	Grade II	5/10 (50 %)
	Grade III	1/3 (33 %)
Dedifferentiated chondrosarcoma		11/32 (34 %)
Mesenchymal chondrosarcoma		0/18 (0 %)
Clear cell chondrosarcoma		1/20 (5 %)
Total		63/187 (34 %)

## Discussion

The aim of this study was to investigate whether the IGF pathway is a suitable target for therapy in chondrosarcoma. Heterogeneous expression of IGF1R, IR, IGF2R, IGF1, IGF2, IRS1 and IGFBP3 was seen, both at the mRNA and protein levels, in chondrosarcoma cell lines. This indicates that essential IGF pathway components are present in cultured chondrosarcoma cells. Furthermore, detection of phosphorylated IRS1 in two out of three chondrosarcoma cell lines demonstrates that the IGF pathway is active in a subset of chondrosarcoma cell lines. In the cell lines with phosphorylated IRS1, IGF pathway inhibition decreased phosphorylated Akt levels and increased IGF1R expression; the latter suggests activation of a feedback loop, which is further supported by the downregulation of IGF1R expression by IGF1 treatment. However, this did not influence the amount of phosphorylated S6, which is located further downstream in the PI3K/Akt/mTOR pathway. Furthermore, the activated MAPK levels were not affected by IGF pathway stimulation or inhibition, demonstrating that activity of the downstream targets is not dependent on IGF1R signalling.

In line with these findings, we demonstrate that despite activity of the pathway, IGF1R signalling is not essential for chondrosarcoma cell survival. Treatment with three different IGF1R/IR inhibitors does not have an effect on chondrosarcoma cell viability, irrespective of apparent pathway activity and stimulation with IGF1. Chondrosarcoma cell line OUMS27 was previously shown to be sensitive to IGF1R/IR inhibition by Zhang et al. [13]. It is difficult to explain the discrepancy with the current study, as OSI-906 is a derivate of the IGF1R inhibitor used by Zang et al. with similar target potency [41]. Moreover, we performed these assays at multiple cell densities, passage numbers and IGF1R/IR inhibitors (data not shown).

IGF1R signalling is involved in resistance to cytotoxic drugs in certain cancers [40]. Since chondrosarcoma is resistant to chemotherapy, we explored a possible role of the IGF1R/IR pathway in chemoresistance. Doxorubicin reduced cell viability in a dose dependent manner; however, OSI-906 did not further inhibit cell viability in this cell line model. These results do not support a key role of the IGF pathway in chondrosarcoma cell survival and chemoresistance.

Our study could not confirm a role for the IGF pathway in chondrosarcoma cell migration. In contrast to the study from Wu et al., showing that IGF1 induced chondrosarcoma migration was inhibited by an IGF1R antibody [23], we chose not to pretreat the chondrosarcoma cells with IGF1 and not to use medium supplemented with IGF1 only as chemoattractant, thereby better mimicking the *in vivo* situation. These experimental differences might explain the difference in our findings.

Strikingly, we detected high expression of the IGF1R in chondrosarcoma cell lines compared to primary tumours. Moreover, we show that each of four patients with matched cell lines and primary tumours had strong membranous IGF1R expression in the cultured cells compared to absent or very weak expression in the corresponding primary tumour. The finding that cell lines are insensitive to IGF1R inhibition despite their high IGF1R expression is in line with the results from the study by Schwartz et al., which described absence of a correlation between IGF1R expression levels and responsiveness to an IGF1R targeting antibody [18]. This series included 38 chondrosarcomas of which 53 % had immunohistochemical staining with an IGF1R antibody [18]. Therefore, we did not anticipate to find weak (34 %) or no expression (66 %) in our cartilage tumour series. The discrepancy between our results and the study from Schwartz et al. can likely be explained by use of another antibody. Lack of reproducibility is a well described phenomenon in preclinical studies with antibodies [42, 43]. Our study further suggests that IGF1R expression is lower in clear cell chondrosarcoma and mesenchymal chondrosarcoma compared to the other cartilage tumours. However, as the staining is very weak in the samples scored positive and IGF1R expression levels do not correlate with responsiveness to IGF1R targeting antibodies, we do not think this difference in IGF1R expression has clinical significance. Furthermore, we did not see a difference in sensitivity to IGF1R inhibition between the mesenchymal, dedifferentiated and conventional chondrosarcoma cell lines included in this study.

Increased activity of the IGF pathway is implicated in several other cancers [12] including other bone tumours [44]. In Ewing sarcoma, IGF binding protein 3 (IGFBP3) is downregulated by the EWSR1-FLI1 fusion gene [45], activating the IGF pathway [46]. Recently, aberrant expression of IGF pathway members was described in osteosarcomas and OSI-906, a dual inhibitor of the IGF1R and the IR, inhibited proliferation in 3 out of 4 osteosarcoma cell lines with IC_50_ values within the therapeutic range [24].

Clinical trials to test the safety and efficacy of IGF1R antibodies, sometimes in combination with an mTOR inhibitor, have been performed in sarcoma patients [47], but only two trials enrolled chondrosarcoma patients [18, 44]. In the study described by Olmos et al. one myxoid chondrosarcoma was included, which showed a small decrease in tumour size upon IGF1R inhibition [44]. It is unclear whether this was an extraskeletal myxoid chondrosarcoma or a chondrosarcoma of bone. In addition, 1 of 17 chondrosarcoma patients showed partial response to Cixutumumab (IGF1R antibody) and Temsirolimus (mTOR inhibitor), as described by Schwarz et al. [18]. In future studies, dual inhibitors of both the IGF1R and the IR are preferably chosen because it has been shown in osteoblasts [48] and Ewing sarcoma cells [49] that cells can circumvent inhibition of IGF1R by increasing IR signalling.

## Conclusions

In summary, the results of this study demonstrate that although chondrosarcoma cell lines have high IGF1R expression and activation of downstream targets, inhibition of IGF1R/IR signalling does not affect chondrosarcoma proliferation, migration and chemoresistance. Therefore, we conclude that there is no convincing preclinical rationale for using IGF1R/IR inhibitors in the treatment of chondrosarcoma.

## Abbreviations

ACT, Atypical cartilage tumour; CIM, Cell Invasion and Migration; FBS, Fetal bovine serum; FFPE, formalin-fixed, paraffin-embedded; HRP, horseradish peroxidase; IGF, Insulin-like growth factor; IGF1R, Insulin-like growth factor receptor; IGFBP, Insulin-like growth factor binding protein; IP, Immunoprecipitation; IR, Insulin receptor; IRS, Insulin receptor substrate; MAPK, Mitogen-activated protein kinases; TMA, Tissue microarray

## Additional files

Additional file 1: Table S1. Properties of the IGF1R inhibitors used in this study as stated by the manufacturer. (DOCX 14 kb) Additional file 2: Table S2. Primers sequences (5’→3’) and positive control tissues. (DOCX 13 kb) Additional file 3: Figure S1. Addition of IGF1 to the medium does not influence chondrosarcoma cell viability. Cells were treated with RPMI 1640 with 10 % FBS, in the presence of absence of IGF1 (50 ng/ml). (PDF 863 KB) Additional file 4: Figure S2. The IGF1R antibody #3018 is more specific than the IGF1R antibody #3027. A: colon tissue stained with IGF1R antibody #3018 (1:1000). B: colon tissue stained with IGF1R antibody #3027 (1:250). Black bars represent 50 μm. C: Western blot comparing IGF1R antibody #3018 (left) and IGF1R antibody #3027 (right) using four chondrosarcoma cell line lysates. With the IGF1R antibody #3027, a strong extra band is observed. (PDF 346 KB) Additional file 5: Figure S3. Positive controls for IGF1R immunohistochemistry. A: colon, B: decalcified colon, C: positive control on TMA (liver). Black bars represent 200 μm. (PDF 0.99 MB) Additional file 6: Figure S4. IGF1R is minimally expressed in primary tumours in contrast to the high expression detected in chondrosarcoma cell lines when derived from the same tumour. IGF1R expression in CH2879 (A-B), L3252B (C-D) and L2975 (E-F) cell lines and primary tumours, respectively. Black bars represent 50 μm. (PDF 913 kb)
