# Supplementary material for: No preclinical rationale for IGF1R directed therapy in chondrosarcoma of bone
Source: BMC Cancer. 2016 Jul 14;16:475. doi: 10.1186/s12885-016-2522-8 (PMC4946092; doi:10.1186/s12885-016-2522-8)
Supplement: Additional file 1: Table S1. — Properties of the IGF1R inhibitors used in this study as stated by the manufacturer. (DOCX 14 kb) [file 12885_2016_2522_MOESM1_ESM.docx]

| **Inhibitor** | **Mechanism of inhibition** | **IC50 IGF1R** | **IC50 IR** | **Other targets** |
| --- | --- | --- | --- | --- |
| OSI-906 | Conformational change by interaction with C-helix | 35 nM | 75 nM |  |
| NVP-ADW742 | ATP-competitive | 170 nM | >16-fold less potent than IGF1R | Little activity to HER2, PDGFR, VEGFR-2, Bcr-Abl and KIT |
| GSK1838705A | ATP-competitive | 2nM | 1.6 nM | Modestly potent to ALK, RSK1 and JNK3 |
