# Supplementary material for: No preclinical rationale for IGF1R directed therapy in chondrosarcoma of bone
Source: BMC Cancer. 2016 Jul 14;16:475. doi: 10.1186/s12885-016-2522-8 (PMC4946092; doi:10.1186/s12885-016-2522-8)
Supplement: Additional file 2: Table S2. — Primers sequences (5’→3’) and positive control tissues. (DOCX 13 kb) [file 12885_2016_2522_MOESM2_ESM.docx]

| **Gene** |  | **+ control tissue** | **Forward primer** | **Reverse primer** |
| --- | --- | --- | --- | --- |
|  |  |  |  |  |
| IGF1 |  | Prostate | GCTCTTCAGTTCGTGTGTGG | ATCCACGATGCCTGTCTGAG |
| IGF1R |  | U2OS | GGAGTCTTCACCACTTACTCGG | CCATGACGAAGCGAAGGACT |
| IGF2 |  | Placenta | GACACCCTCCAGTTCGTCT | ACAGCACTCCTCAACGATGC |
| IGF2R |  | Hela | GGACTACGACAGAGACCACG | CTGAAGACTTGTGGCCTCCC |
| IGFBP3 |  | Placenta | GCCAGCGCTACAAAGTTGAC | TGTCTTCCATTTCTCTACGGCA |
| IR |  | Colon | CCCAATGGTCTGATCGTGCT | CGAAGTGCTTGCGGGAGA |
