# Supplementary figures and images for: No preclinical rationale for IGF1R directed therapy in chondrosarcoma of bone
Source: BMC Cancer. 2016 Jul 14;16:475. doi: 10.1186/s12885-016-2522-8 (PMC4946092; doi:10.1186/s12885-016-2522-8)

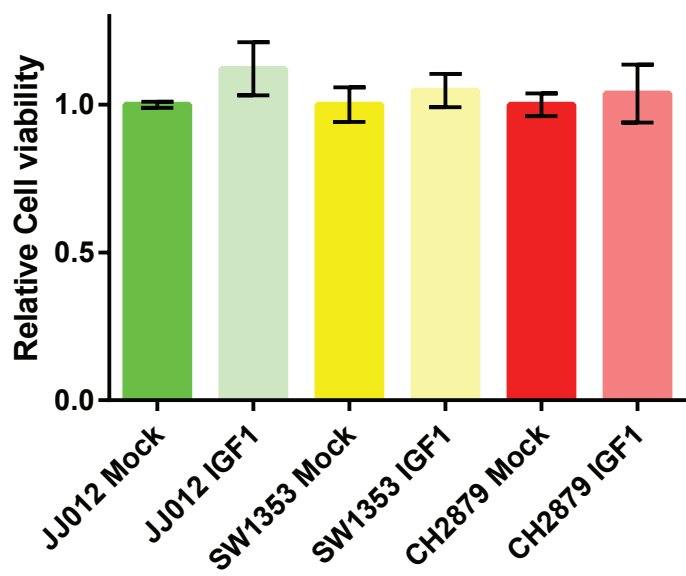

Supplement: Additional file 3: Figure S1. — Addition of IGF1 to the medium does not influence chondrosarcoma cell viability. Cells were treated with RPMI 1640 with 10 % FBS, in the presence of absence of IGF1 (50 ng/ml). (PDF 863 KB) [file 12885_2016_2522_MOESM3_ESM.pdf]

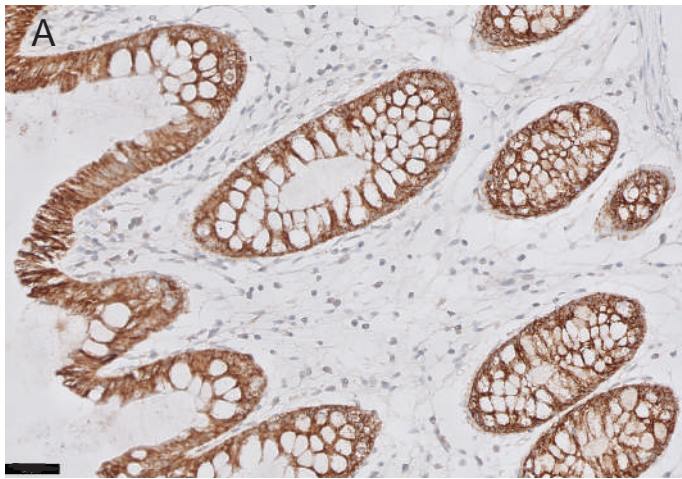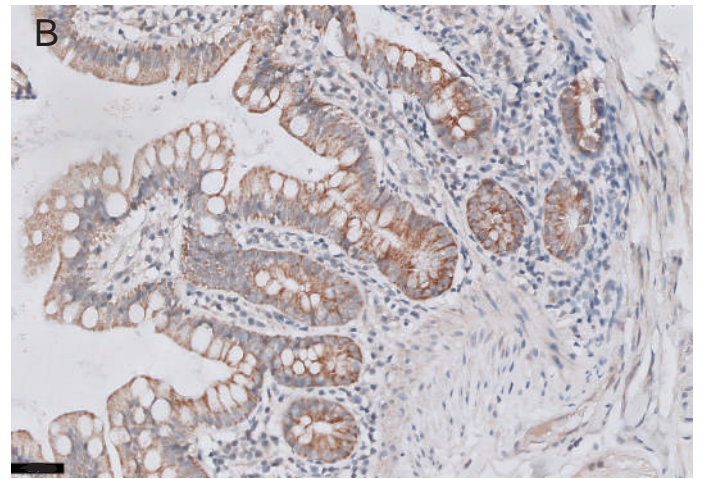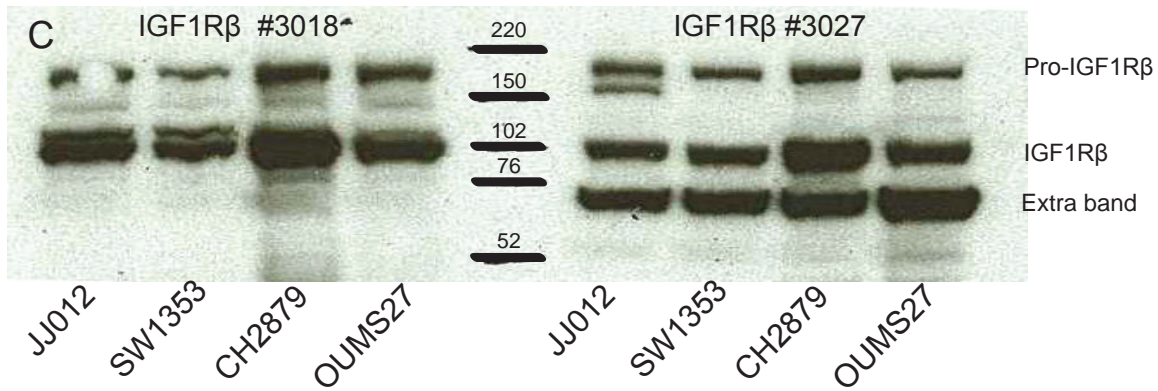

Supplement: Additional file 4: Figure S2. — The IGF1R antibody #3018 is more specific than the IGF1R antibody #3027. A: colon tissue stained with IGF1R antibody #3018 (1:1000). B: colon tissue stained with IGF1R antibody #3027 (1:250). Black bars represent 50 μm. C: Western blot comparing IGF1R antibody #3018 (left) and IGF1R antibody #3027 (right) using four chondrosarcoma cell line lysates. With the IGF1R antibody #3027, a strong extra band is observed. (PDF 346 KB) [file 12885_2016_2522_MOESM4_ESM.pdf]

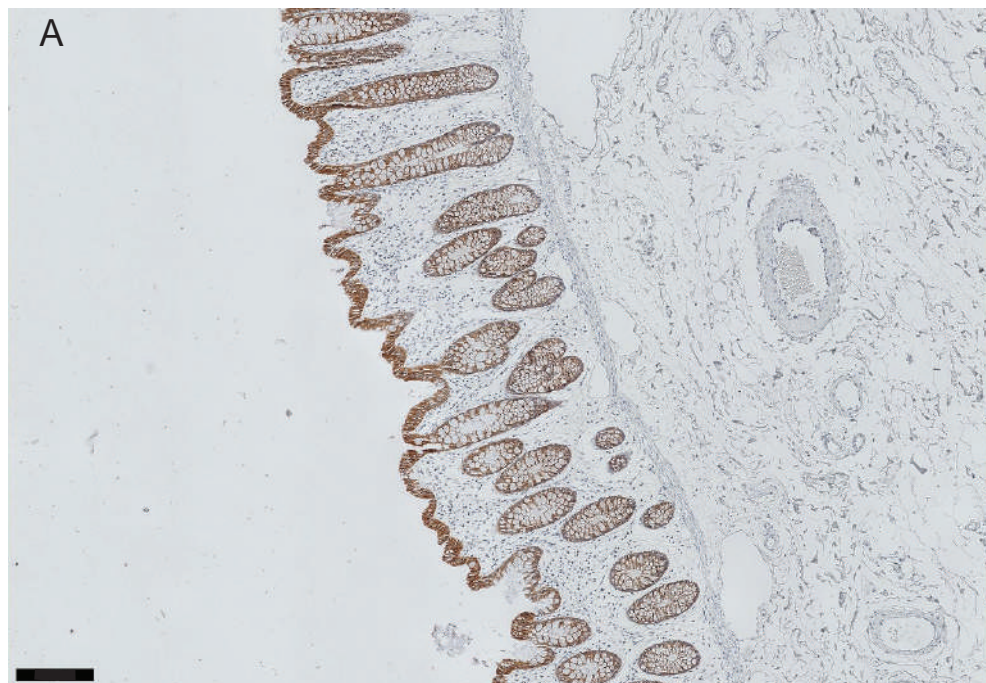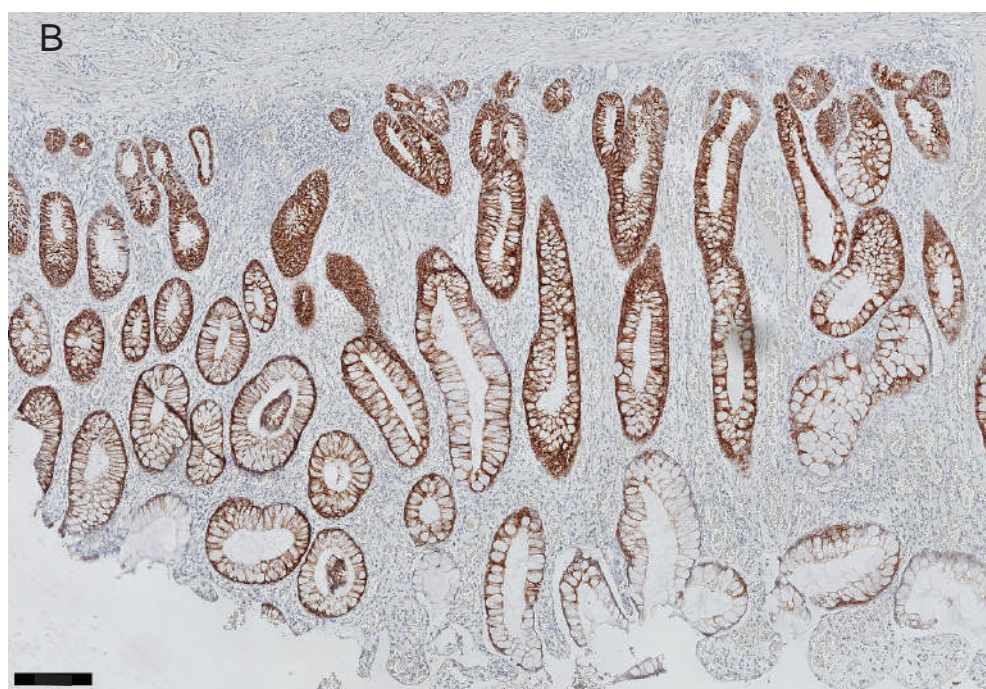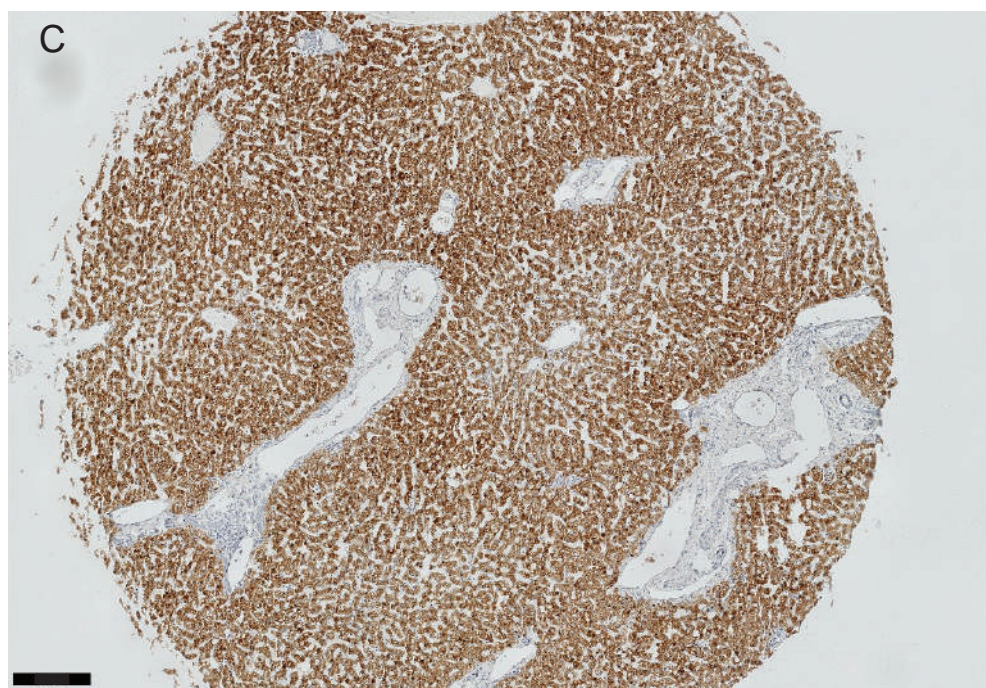

Supplement: Additional file 5: Figure S3. — Positive controls for IGF1R immunohistochemistry. A: colon, B: decalcified colon, C: positive control on TMA (liver). Black bars represent 200 μm. (PDF 0.99 MB) [file 12885_2016_2522_MOESM5_ESM.pdf]

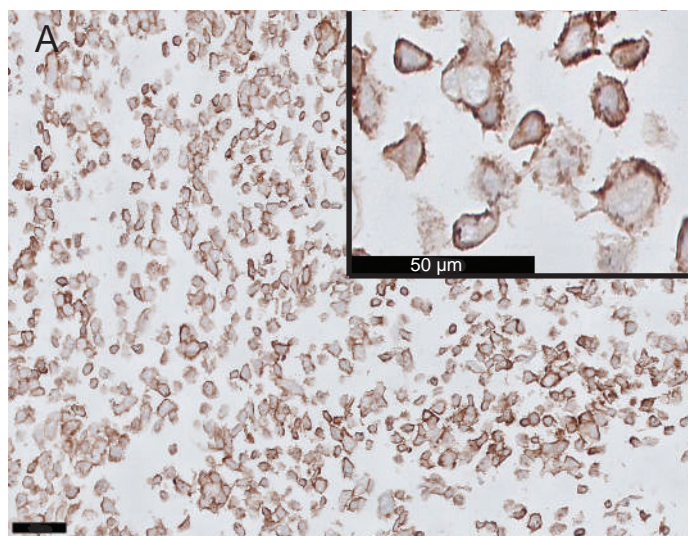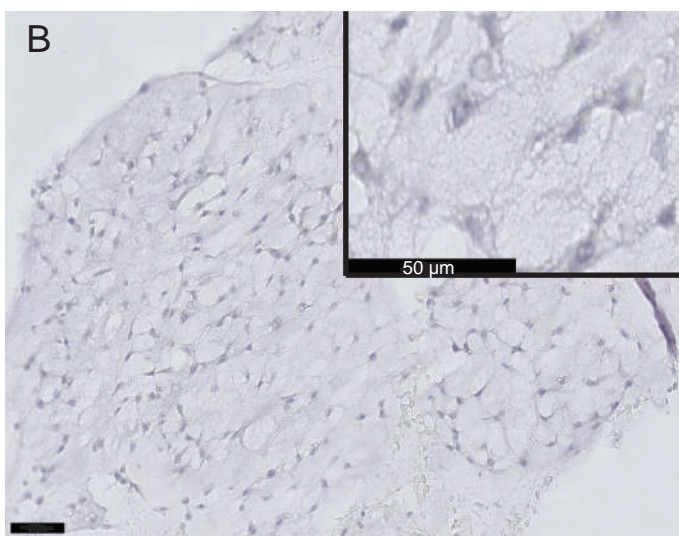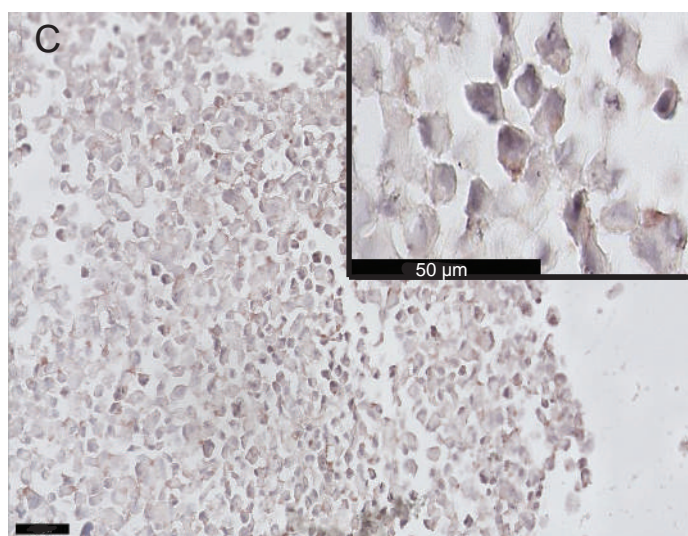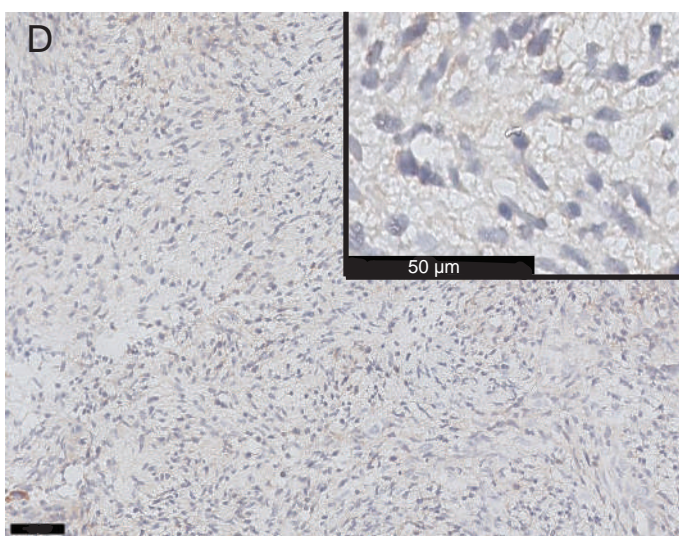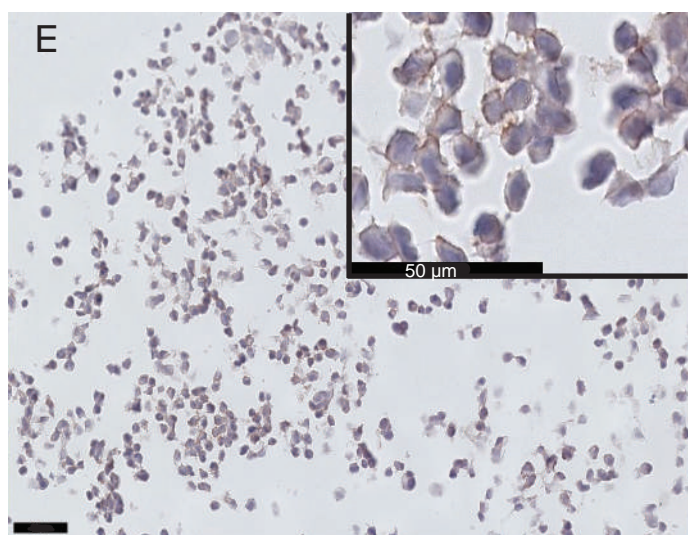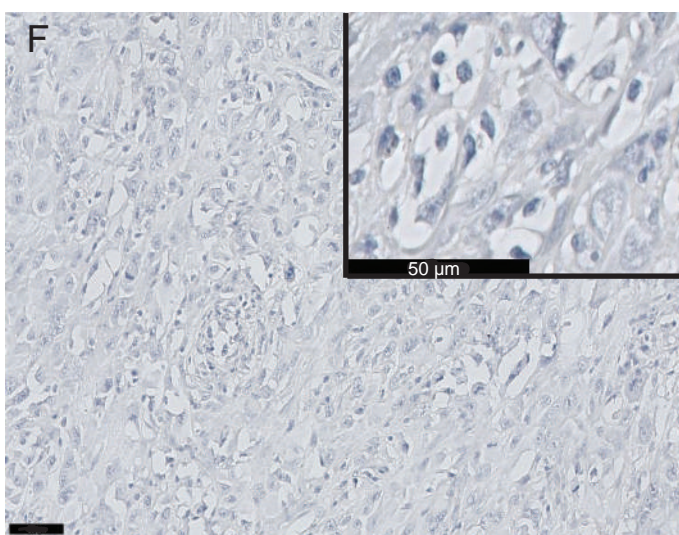

Supplement: Additional file 6: Figure S4. — IGF1R is minimally expressed in primary tumours in contrast to the high expression detected in chondrosarcoma cell lines when derived from the same tumour. IGF1R expression in CH2879 (A-B), L3252B (C-D) and L2975 (E-F) cell lines and primary tumours, respectively. Black bars represent 50 μm. (PDF 913 kb) [file 12885_2016_2522_MOESM6_ESM.pdf]
